# Experimental Determination of the Ionization Constant of *N*,*N*‑diethylhydroxylamine (DEHA) from 25 to 250 °C at 15 MPa

**DOI:** 10.1021/acs.jced.6c00121

**Published:** 2026-07-30

**Authors:** Sreedhanya Swaroop, Swaroop Sasidharanpillai, Peter R. Tremaine

**Affiliations:** Department of Chemistry, 3653 University of Guelph, 50 Stone Rd. E., Guelph, Ontario N1G 2W1, Canada

## Abstract

Ionization constants, *K*_a,H_, for *N*,*N*-diethylhydroxylamine (DEHA) have been measured under hydrothermal conditions, from 25 to 250 °C at 15 MPa, using the thermally stable colorimetric pH indicator acridine. The measurements were carried out by UV–visible spectroscopy using a high-temperature, high-pressure titanium flow cell with sapphire windows. The use of flow minimized the effects of thermal decomposition because the data could be collected rapidly with short equilibration times. NMR and Raman spectroscopic analysis of the quenched experimental solutions from 250 °C showed that the extent of DEHA decomposition in the experimental solution was minimal at the highest temperature studied. These are the first experimental values for the ionization constant of DEHA above 25 °C that have been reported. The experimental values of p*K*_a,H_ for DEHA were fitted using the simplified form of the extended van’t Hoff equation for an isocoulombic reaction and with Mesmer’s “Density” model to generate thermochemical parameters suitable for the EPRI MULTEQ chemical equilibrium model and other similar software used to model water chemistry in nuclear reactor secondary coolant systems and in fossil steam generating systems.

## Graphical abstract

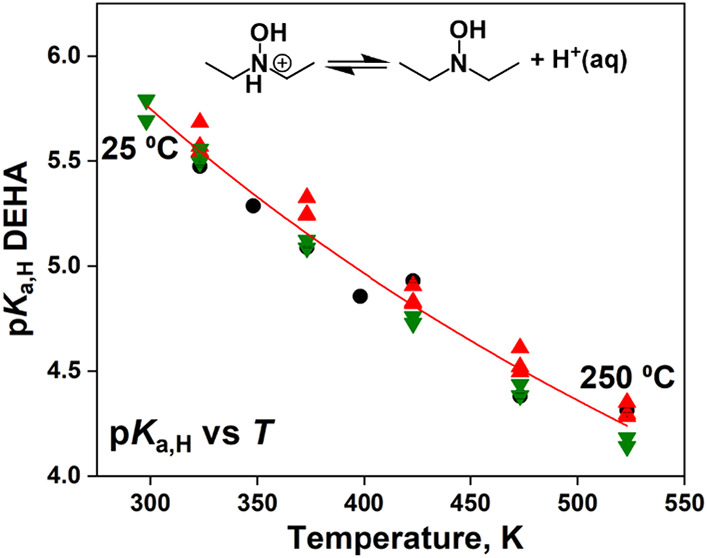

## 1 Introduction

The chemistry control for the secondary coolant loop of CANDU and PWR nuclear power plants, and fossil power plant steam generating systems, utilizes volatile amines or ammonia as pH control agents along with hydrazine as an oxygen scavenger.^1–5^ However, hydrazine has been identified as a potential carcinogenic substance and efforts are in place to replace it with less hazardous reducing agents.^5–7^ *N*,*N*-diethylhydroxylamine (DEHA) is being evaluated as a candidate of particular interest. Extensive studies have been carried out to understand the kinetics of DEHA’s reactions with oxygen,^6,8^ and the effects of its thermal decomposition products on the corrosion of carbon steel.^9,10^

To model DEHA’s effectiveness as a secondary coolant additive, thermochemical parameters for aqueous DEHA are required at temperatures from 25 to 275 °C. The ionization equilibrium of DEHA takes place according to Reaction (1). The ionization constant of DEHA is known only at 25 °C.^11^ Indeed, there are no known values for the ionization constants for any hydroxylamine in water at temperatures above 35 °C.



1

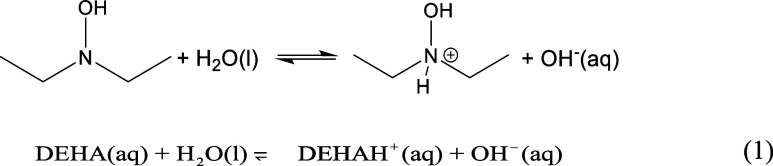

One of the key parameters required to model amine chemistry is the ionization constant. Experimental strategies for measuring the ionization constants of amines under steam-generator conditions have been reviewed by Tremaine et al.^12^ and have been used with success for amines other than hydroxylamines. Most commonly, they are determined directly as the dissociation constant for Reaction ([Disp-formula eq1]), *K*_b,OH_ by high temperature potentiometric pH titrations in hydrogen concentration cells, UV–visible spectroscopy or by AC conductivity. An alternative is to calculate *K*_b,OH_ vs *T* from accurate standard partial molar enthalpies of ionization at 25 °C, Δ_ion_ *H*_298_^o^, and temperature-dependent heat capacities of reaction, Δ_ion_ *C*_*p,* *T*_^o^.^13^ There are no known values of Δ_ion_ *H*_298_^o^ or Δ_ion_ *C*_*p* *,T*_^o^ for DEHA in the literature.

Acid–base ionization constants for species which lack a characteristic absorption band in the UV–visible spectrum can be measured using thermally stable colorimetric pH indicators in UV–visible spectrophotometric flow cells.^14–17^ Several indicators have been identified that can be used in aqueous systems at temperatures as high as 400 °C, with a precision of ±0.1 pH unit.^14,17,18^

The work described here measured the ionization constants of DEHA, *K*_a,H_ vs *T*, from ambient up to steam generator conditions by UV–visible spectroscopy using a thermally stable pH indicator. In this method, the equilibrium molality of H^+^ is determined by measuring the relative concentration of each form of the appropriate pH indicator in DEHA/DEHAHCl buffer solutions at temperatures up to 250 °C at a controlled pressure of 15 MPa. The ionization constant is then determined from the Henderson–Hasselbalch equation,^19^ log *K*_a,H_ = log *m*_H^+^_ + log *m*_DEHA_/*m*_DEHAH^+^_ and log *K*_b,OH_ = log *K*_w_ – log *K*_a,H_. The flow systems described by Ehlerova et al.^14^ and Bulemela et al.^17^ allow quantitative UV–visible spectra to be measured up to 350 °C and 20 MPa, using platinum and titanium cells for reducing and oxidizing conditions, respectively. Because it is a flow system, such measurements have the advantage of relative ease and speed, including a residence time that minimizes thermal decomposition of the sample. Ionization constants calculated from the measured equilibrium pH were fitted with the temperature-dependent expressions like the extended van’t Hoff equation and Mesmer “Density” model.^20^

## 2 Experimental Methods

### 2.1 Chemicals

N,N-Diethylhydroxylamine (DEHA) (Millipore Sigma, ≥98%) was used as received to prepare stock solutions by mass. DEHA stock solution and DEHAHCl buffer solutions were prepared inside an argon glovebox to prevent atmospheric CO_2_ contamination. The colorimetric indicator, acridine (Sigma-Aldrich 97%), was used without further purification and was stored as the solid in a dark brown bottle to minimize photodecomposition. Concentrated solutions of NaOH (Fisher Scientific, 50% w/w) and HCl (2 N ± 0.08 N, Spectrum Chemical Fisher Scientific) were used to prepare stock solutions. The NaOH solutions were standardized by triplicate titration using potassium hydrogen phthalate (Sigma-Aldrich, 99.95%) that had been dried at 110 °C for 2 h prior to use. The HCl solutions were standardized by titration against tris­(hydroxymethyl)­aminomethane (Sigma-Aldrich, 99.9%) which had been previously dried to constant mass at 110 °C. Specifications of the chemicals used in this study are reported in Table 1. The DEHA solutions were standardized by titration with the previously standardized HCl. Nanopure water (resistivity >18 MΩ·cm) was degassed prior to the preparation of all the solutions.

**Table 1:** Specification of Chemicals Used in This Study

chemicals	molecular formula	molar mass (g mol^–1^)	supplier	CAS number	purity
*N*,*N*-Diethylhydroxylamine	(CH_3_CH_2_)_2_NOH	89.14	Sigma-Aldrich	3710–84–7	≥98%
Acridine	C_13_H_9_N	179.22	Sigma-Aldrich	260–94–6	97%
Sodium Hydroxide Solution (50% w/w)	NaOH	39.997	Thermo Fisher Scientific	1310–73–2, 7732–18–5	99.91%
Hydrochloric Acid 2.0 N Solution	HCl	36.46	Spectrum Chemical	7647–01–0, 7732–18–5	99.99%
Potassium hydrogen phthalate, primary standard	C_8_H_5_KO_4_	204.222	Sigma-Aldrich	877–24–7	99.95%
Tris(hydroxymethyl)aminomethane	NH_2_C(CH_2_OH)_3_	121.14	Sigma-Aldrich	77–86–1	≥99.9%

Because acridine is quite insoluble, standard solutions for these experiments were prepared from stock solutions of the chloride salt. Each ∼10^–4^ mol·kg^–1^ stock solution of AcrH^+^ indicator was prepared fresh on the day before each set of experiments by dissolving ∼5 mg acridine in 0.6 or 0.3 mol·kg^–1^ standardized solution of HCl. Aliquots of the stock indicator solutions were then transferred quantitatively into two Nalgene bottles. It was then diluted to prepare ∼10^–5^ mol·kg^–1^ standard solution of the AcrH^+^ acid extreme. More of a 0.3 mol·kg^–1^ standard solution of NaOH, was added prior to dilution to prepare ∼10^–5^ mol·kg^–1^ standard solution of the Acr base extreme. Aliquots of the same mass of stock indicator solutions, neutralized using NaOH, were added to each DEHA/DEHAH^+^ buffer solutions to obtain the required experimental buffer ratios.

At each step, the masses of the solutions and added water were recorded to calculate the compositions of experimental solutions. This approach avoids the need to know the exact molality of indicator in the initial stock solution by quantitatively tracking the relative molality of total acridine through each of the subsequent dilution steps.

### 2.2 UV–Visible Spectrometer and High-Temperature Titanium Flow Cell

Equilibrium constants were measured in a high temperature, high-pressure spectroscopic flow system similar to that described by Trevani et al.,^21^ with some modifications. The schematic diagram of the modified high-pressure flow cell is shown in Figure 1.

**Figure 1:**
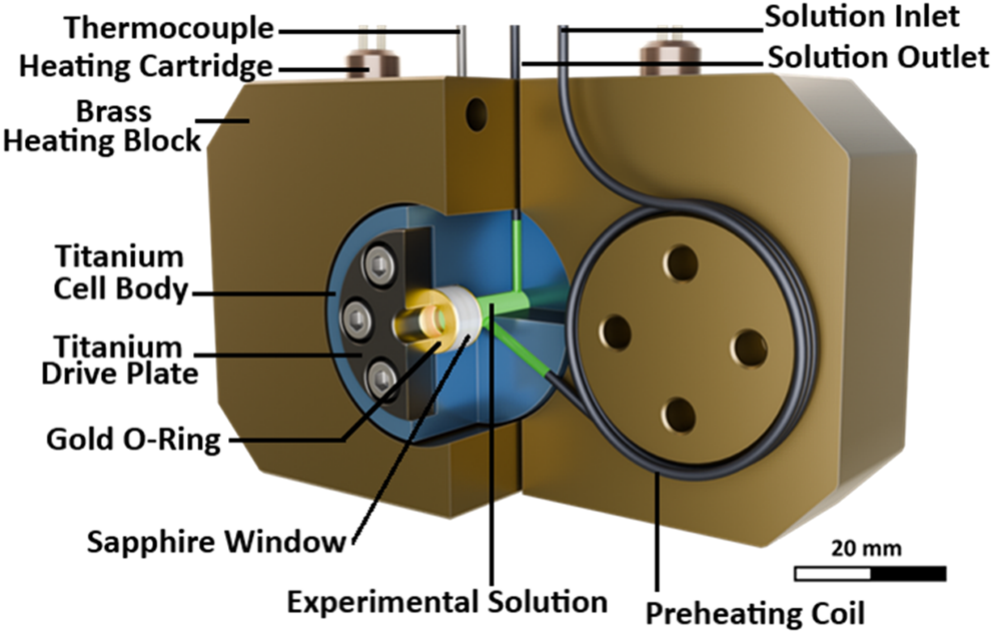
Schematic diagram of the high-temperature titanium UV–visible flow cell.

The low volume cell (0.33 cm^3^ internal volume) is made of titanium and sapphire windows sealed with gold seals. The cell was secured in a three-piece brass oven and heated using two Chromalux CIR-20203 cartridge heaters. Before reaching the cell, the injected solutions pass through a 30 cm preheater coil of small diameter (0.508 mm inner diameter) titanium high pressure liquid chromatography (HPLC) tubing set within a groove cut in the body of the brass block. The temperature was maintained to within ±1 °C with an Omega CN76000 temperature controller and monitored with a Chromega-Alomega thermocouple inserted directly into the Ti cell body. Under hydrothermal conditions, the experimental solutions were in contact only with the inert sapphire windows, titanium body of cell and preheater tubing, and gold seals. The cell-oven system was insulated using ceramic insulation and was mounted in a small aluminum box which is cooled by internal coolant circulation to maintain the spectrophotometer compartment at room temperature. A Varian Cary 50 spectrophotometer (190–1100 nm) with CaryWin UV Scan application software was used to record the absorption spectra.

The sample was injected into the cell using a sample injection system as shown in Figure 2. A Gilson 305 HPLC piston pump, equipped with a Gilson manometric module (Model 805) was used to pump water through a six-port titanium valve fitted with a 20 cm^3^ sample injection loop, on through a titanium preheating loop and into the cell. A second loop placed downstream of the cell allowed the solution to be sampled after it had passed through the cell. Pressure was controlled by a P-880 high pressure adjustable back-pressure regulator (IDEX Health and Science).

**Figure 2:**
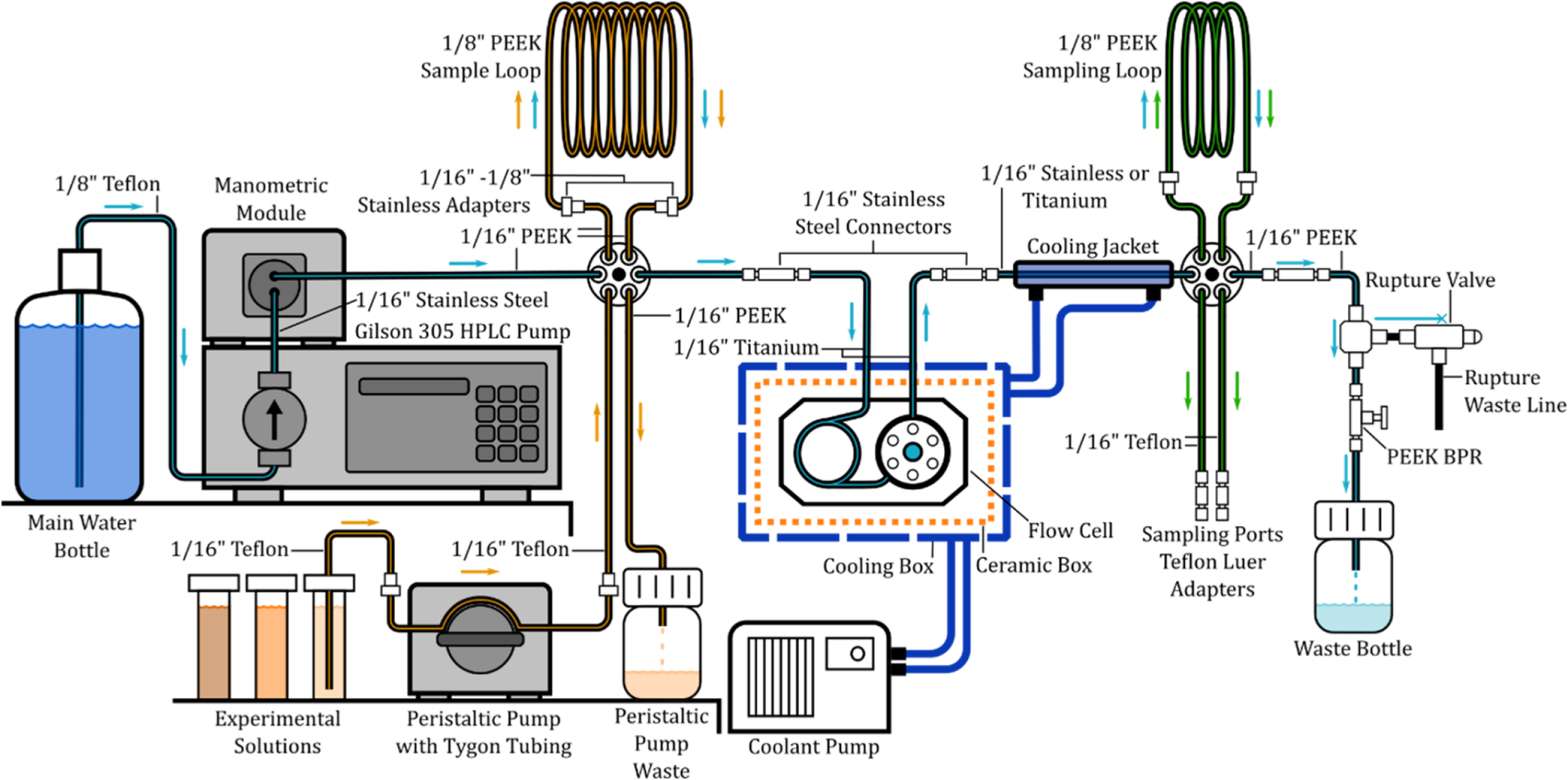
Schematic diagram of the flow injection system.

### 2.3 Methods

#### 2.3.1 Ionization Constants

All solutions were degassed by purging argon prior to injection into the titanium flow cell. The high-pressure injection system was operated at a constant flow rate of 0.1 mL·min^–1^ up to 150 °C and a flow rate of 1.0 mL·min^–1^ was chosen above 150 °C to minimize the effects of thermal decomposition. Flow rate tests using the acridine base extreme solution and other thermally stable indicators showed that the absorbance spectra were identical to within ±1% at flow rates up to and above 1 mL·min^–1^ at temperatures as high as 300 °C, confirming that they were in thermal equilibrium with the brass block. The pressure was fixed at 15 MPa in all experiments to guarantee a single liquid phase for the solutions. Experimental solutions (∼20 mL) were introduced into the titanium cell in the UV–visible spectrophotometer by means of a high-pressure liquid chromatography injection loop. Spectra were collected in triplicate at wavelengths between 200 and 600 nm and were averaged to minimize the noise. Spectra of solutions without the indicator were also recorded under the same conditions for baseline correction. Preliminary calculations suggested that two thermally stable indicators could be used, acridine at temperatures from 25 to 250 °C and β-naphthoic acid from 50 to 150 °C (Figure S1). However, all the measurements were done with acridine as it was found to be the suitable indicator for the full range of experimental temperatures.

#### 2.3.2 Thermal Decomposition Studies by Raman and NMR Spectroscopy

The DEHA buffer solutions were examined by ^1^H, ^13^C NMR and Raman spectroscopy before and after injection to ensure the purity of initial solutions and to ensure that no thermal decomposition has taken place. In the course of the UV–visible studies at 250 °C, the DEHA/DEHAH^+^ buffer solutions were run at flow rates of 1 and 0.1 mL·min^–1^ to achieve different residence times. The quenched solutions were collected downstream of the flow cell during these experiments for analysis by ^1^H and ^13^C NMR spectroscopy using a Bruker 400 MHz Nuclear Magnetic Resonance spectrometer. Samples of the original unheated solution, and quenched samples of heated solutions that had passed through the cell, were spiked with sodium 2,2-dimethyl-2-silapentane-5-sulfonate (DSS) as an NMR internal standard for quantification and analysis by ^1^H NMR. Sample analysis by Raman spectroscopy was carried out in quartz tubes in the macrochamber of the custom-made Horiba LabRAM HR 800 spectrometer. The Raman spectra were measured using 532 nm laser excitation and 600 lines/mm grating.

## 3 UV–Visible Spectroscopic Analysis

The equilibrium constant for ionization of DEHA according to Reaction 1 is

2 Kb,OH=mDEHAH+mOH−mDEHAmHO2γDEHAH+γOH−γDEHAγHO2

The ionization reaction can also be written in the charge symmetric (isocoulombic) form as

3 $${\mathrm{DEHAH}}^{+}\left(\mathrm{aq}\right)⇌{\mathrm{DEHA}\left(\mathrm{aq}\right)+H}^{+}\left(\mathrm{aq}\right)$$

The equilibrium constant for this reaction is

4 $$K_{a,H}=\frac{m_{\mathrm{DEHA}}m_{H^{+}}}{m_{{\mathrm{DEHAH}}^{+}}}\frac{\gamma_{\mathrm{DEHA}}\gamma_{H^{+}}}{\gamma_{{\mathrm{DEHAH}}^{+}}}$$

where, *m_i_* is the molality and γ*_i_* is the activity coefficients of the species, respectively. The activity coefficient of the neutral species, γ_DEHA_, was assumed to be unity.

Thermally stable colorimetric indicators have been used in UV–visible flow cells to determine ionization constants of acids and bases under hydrothermal conditions.^18,22–24^ The method involves determination of pH from the spectrum of a trace amount of suitable indicator added to the buffer solution of the analyte. The measurement of indicator spectra in strong acid and base medium under the same conditions gives spectra for the acidic and basic forms of the indicator and the spectrum of indicator in buffer solution is a linear combination of the spectra of the two forms of the indicator, from which the equilibrium pH can be calculated. Acridine was used as the indicator in the present study. The ionization reaction of the indicator acridine was represented as

5 $${\mathrm{AcrH}}^{+}\left(\mathrm{aq}\right)⇌\mathrm{Acr}\left(\mathrm{aq}\right)+H^{+}\left(\mathrm{aq}\right)$$

and the equilibrium constant as

6 $$K_{{\mathrm{AcrH}}^{+}}=\frac{m_{\mathrm{Acr}}m_{H^{+}}}{m_{{\mathrm{AcrH}}^{+}}}\frac{\gamma_{\mathrm{Acr}}\gamma_{H^{+}}}{\gamma_{{\mathrm{AcrH}}^{+}}}$$

The temperature dependent ionization constants for acridine (*K*_AcrH__^+^_) are based on the expression by Ryan et al.,^23^ given as

7 $$\ln K_{{\mathrm{AcrH}}^{+}}=-12.43-3663.04\left(\frac{1}{T}-\frac{1}{T_{r}}\right)-\frac{15874.31}{T}\left(\frac{1}{\varepsilon_{T}}-\frac{1}{\varepsilon_{r}}\right)$$

where *T* is temperature in Kelvin, *T*_r_ = 298.15 K, ε_*T*_ is the static dielectric constant of water at temperature *T* and ε_r_ = 78.38011 is the static dielectric constant of water at *T*_r_ = 298.15 K.^25^

Equation ([Disp-formula eq6]) can be rearranged to determine the hydrogen ion molality as

8 $$m_{H^{+}}=K_{\mathrm{Acr}H^{+}}\frac{m_{\mathrm{Acr}H^{+}}}{m_{\mathrm{Acr}}}\frac{\gamma_{\mathrm{Acr}H^{+}}}{\gamma_{H^{+}}}$$

The activity coefficient of neutral species, γ_Acr_, was assumed to be unity. Substituting eq ([Disp-formula eq8]) into eq ([Disp-formula eq4]) thus yields the expression for the dissociation constant of DEHA as

9 $$K_{a,H}=K_{{\mathrm{AcrH}}^{+}}\frac{m_{{\mathrm{AcrH}}^{+}}\gamma_{{\mathrm{AcrH}}^{+}}}{m_{\mathrm{Acr}}}\frac{m_{\mathrm{DEHA}}}{m_{{\mathrm{DEHAH}}^{+}}\gamma_{{\mathrm{DEHAH}}^{+}}}$$

For dilute solutions at elevated temperatures, the activity coefficient of charged species, γ*_i_*, can be considered independent of the relative compositions of the mixture and dependent only on the total ionic strength.^1,26,27^ The value of the total ionic strength at experimental conditions was calculated to be less than 0.2 mol·kg^–1^ in every buffer solution studied. The activity coefficients were approximated by a “model substance” approach^27^ given by

$$\gamma_{i}={\left(\gamma_{\pm ,NaCl}\right)}^{{z_{i}}^{2}}$$ 10

where *z_i_* is the ionic charge and γ*_i_* is the activity coefficient of species *i* and γ_±, NaCl_ is the mean ionic activity coefficient for NaCl at the same ionic strength. Values of γ_±, NaCl_ were calculated from the Archer equation of state for NaCl.^28^ Thus, (γ_AcrH^+^_/γ_DEHAH^+^_) = 1 because each ion has the same ionic charge and can be approximated in the same way using eq ([Disp-formula eq10]). This allows eq ([Disp-formula eq9]) to be simplified as

11 $$K_{a,H}=K_{{\mathrm{AcrH}}^{+}}\left(\frac{m_{{\mathrm{AcrH}}^{+}}}{m_{\mathrm{Ac}r}}\right)\left(\frac{m_{\mathrm{DEHA}}}{m_{{\mathrm{DEHAH}}^{+}}}\right)$$

Here, the buffer ratios (*m*_DEHA_/*m*_DEHAH^+^_), were taken to be equal to the initial composition of the buffer solutions. The experimental indicator ratios (*m*_AcrH^+^_/*m*_Acr_) were determined by deconvoluting the UV–visible spectra to obtain the amount of acid and basic forms, in which the total absorbance was considered to be the linear combination of the spectra of both forms of the indicator according to eq ([Disp-formula eq12])

12 $$A\left(\lambda \right)=\left[\varepsilon_{{\mathrm{AcrH}}^{+}}\left(\lambda \right)bm_{{\mathrm{AcrH}}^{+}}^{*}+\varepsilon_{\mathrm{Ac}r}\left(\lambda \right)bm_{\mathrm{Acr}}^{*}\right]\rho_{\mathrm{solution}}$$

where A­(λ) is the absorbance at each wavelength; m*_i_ is the molality in terms of mol.(kg of solution)^−1^; *b* is the path length of the cell; and ρ_solution_ is the solution density. The terms ε_AcrH_^+^(λ) and ε_Acr_(λ) are the absorptivities of the indicator in the acid form and its conjugate base. These were determined from independent experiments with indicator solutions in excess of hydrochloric acid and sodium hydroxide, respectively as

13 $$\varepsilon_{{\mathrm{AcrH}}^{+}}\left(\lambda \right)=\frac{A_{\mathrm{acid}}\left(\lambda \right)}{bm_{\mathrm{acid}}^{*}\rho_{\mathrm{acid}}}$$

14 $$\varepsilon_{\mathrm{Acr}}\left(\lambda \right)=\frac{A_{\mathrm{base}}\left(\lambda \right)}{bm_{\mathrm{base}}^{*}\rho_{\mathrm{base}}}$$

where m*_acid_ and m*_base_ represent the molality of AcrH^+^ and Acr in the acidic and basic solutions, respectively. The terms, *A*_acid_(λ), ρ_acid_, *A*_base_(λ) and ρ_base_ are the absorbance and density of the indicator in hydrochloric acid and sodium hydroxide solutions, respectively. Substituting eqs ([Disp-formula eq13]) and ([Disp-formula eq14]) into eq ([Disp-formula eq12]) gives

15 $$\frac{A\left(\lambda \right)}{{b\rho }_{\mathrm{solution}}}=\frac{A_{\mathrm{acid}}\left(\lambda \right)}{bm_{\mathrm{acid}}^{*}\rho_{\mathrm{acid}}}\left(m_{{\mathrm{AcrH}}^{+}}^{*}+\frac{A_{\mathrm{base}}\left(\lambda \right)\rho_{\mathrm{acid}}}{A_{\mathrm{acid}}\left(\lambda \right)\rho_{\mathrm{base}}}D_{\mathrm{In}}m_{\mathrm{Acr}}^{*}\right)$$

where *D*_In_ = m*_acid_ /m*_base_, is the ratio of the total acridine molalities in the acidic and basic solutions. By assuming that the densities are the same in dilute solutions, i.e., ρ = ρ_solution_ = ρ_acid_ = ρ_base_, eq ([Disp-formula eq15]) was simplified as

16 $$A\left(\lambda \right)=A_{\mathrm{acid}}\left(\lambda \right)\frac{m_{{\mathrm{AcrH}}^{+}}^{*}}{m_{\mathrm{acid}}^{*}}+A_{\mathrm{base}}\left(\lambda \right)D_{\mathrm{In}}\frac{m_{\mathrm{Acr}}^{*}}{m_{\mathrm{acid}}^{*}}$$

## 4 Results

### 4.1 UV–Visible Spectra and Thermal Stability Effects

Typical UV–visible spectra for acridine in aqueous HCl (the acid extreme), NaOH (the basic extreme) and in a series of DEHA buffers are shown in Figure 3, at temperatures of 25, 150, and 250 °C. These spectra were measured at a constant flow rate of 0.1 mL·min^–1^ up to 150 °C and at 1.0 mL·min^–1^ above 150 °C. As illustrated in Figure 3, the spectra of acridine in the base and buffer solutions were consistent with one another, showing the isosbestic points noted by Ryan et al.^23^ for AcrH^+^ and Acr corresponding to the values 363 and 373 nm at 25 °C, with minor discrepancies. As was observed in previous studies,^15,29^ the spectra of AcrH^+^ in the acid solution displayed a small but consistent shift to higher absorptivities relative to the base and buffer solutions. Measurements in conventional cuvette cells up to 80 °C did not show this effect. We speculate that the effect is caused by interactions of the dilute acridine solutions with the sapphire windows.^30^

**Figure 3:**
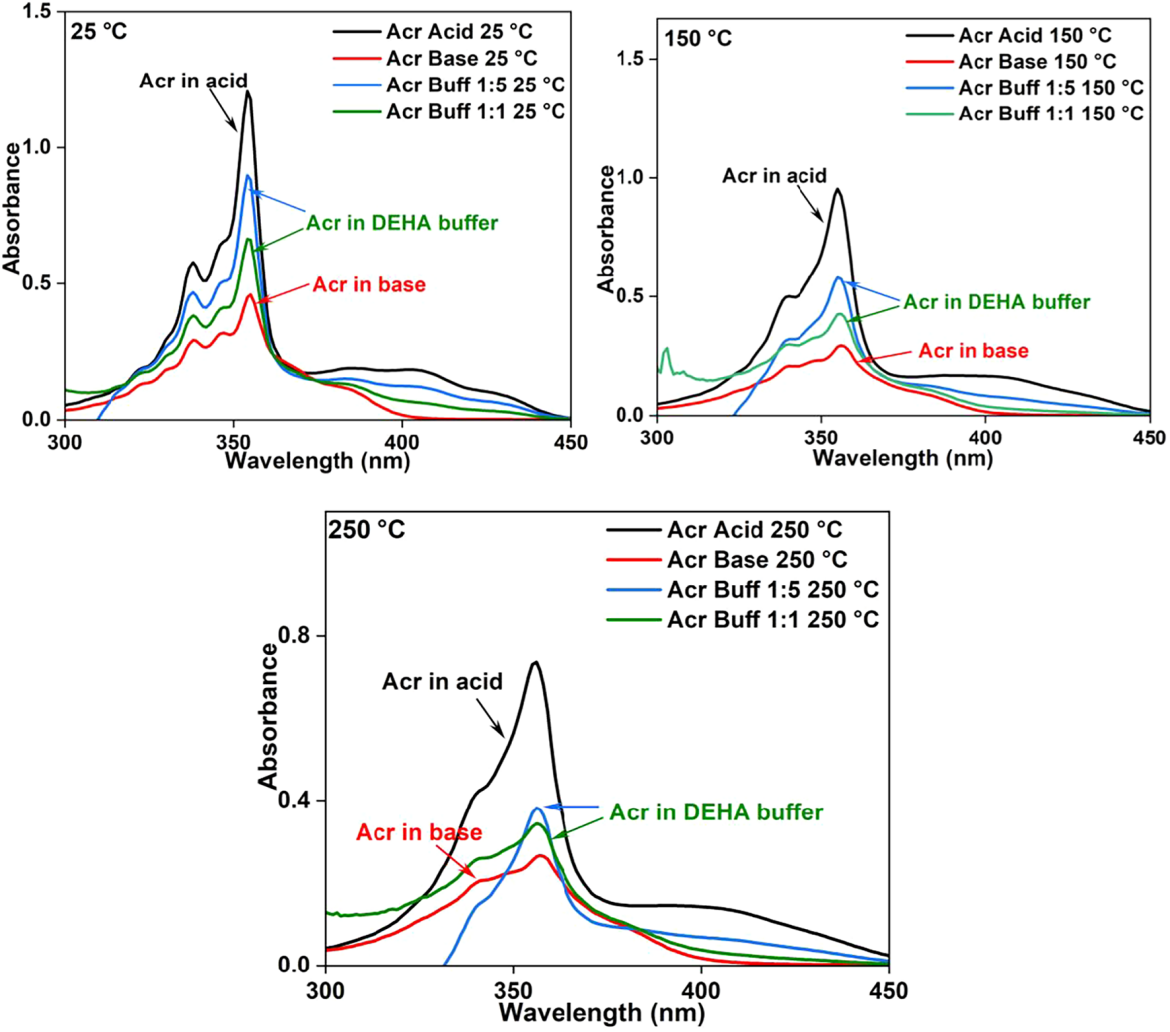
Comparison of the UV–visible absorption spectra of acridine in hydrochloric acid (acid extremum), sodium hydroxide (base extremum), and several DEHA/DEHAH^+^ buffer solutions at temperatures, *t* = 25, 150, and 250 °C and pressure, *p* = 15 MPa. Data between 410 and 435 nm were used for the linear regression of eq ([Disp-formula eq17]).

As noted above, in studies to test for thermal decomposition effects at 225 and 250 °C, the experimental flow rate was set at 0.1 mL·min^–1^ to increase to the residence time of the solution at elevated temperature. The ^1^H and ^13^C NMR spectra of the quenched solutions from these runs are reported as Figures S2 and S3, Supporting Information. Briefly, the intensities of the DEHA peaks in the ^1^H and ^13^C spectra were somewhat reduced, and the appearance of small new peaks indicated the presence of thermal decomposition of products. Based on the integrated ^1^H band areas, the thermal decomposition of DEHA in the UV–visible flow cell at a flow rate of 1.0 mL·min^–1^ is ∼< 7% at 250 °C and is estimated to be minor, (≤2%) at 225 °C. The ^13^C NMR spectra and Raman analysis of the quenched samples are consistent with these results. Full details are reported in Supporting Information.

Detailed analysis of the ^1^H and ^13^C NMR spectra (Figures S4 and S5) of DEHA solutions (0.21 mol·kg^–1^) heated for longer period (1 h) in Teflon pressure vessel at 250 °C showed the presence of diethylamine and methylethylamine, consistent with previous thermal decomposition studies.^7^ Details are given in Supporting Information.

### 4.2 Ionization Constants of DEHA from 25–250 °C

The spectra of acridine in each acid medium corresponds to AcrH^+^, and that in basic medium corresponds to neutral Acr. From Figure 3, it is evident that the spectra in the buffer solutions is a linear combination of spectra in acid and base medium where AcrH^+^ and Acr coexist.

The data analysis was carried out using a simplified form of eq ([Disp-formula eq16]) that takes the form

17 $$A\left(\lambda \right)=A_{\mathrm{acid}}\left(\lambda \right)k+A_{\mathrm{base}}\left(\lambda \right)D_{\mathrm{In}}l$$

where “*k*” and “*l*” are proportionality constant. The indicator ratio was then obtained as

18 $$\frac{k}{l}=\frac{m_{{\mathrm{AcrH}}^{+}}^{*}}{m_{\mathrm{Acr}}^{*}}=\frac{m_{{\mathrm{AcrH}}^{+}}}{m_{\mathrm{Acr}}}$$

The linear regression of eq ([Disp-formula eq17]) to the indicator spectrum for each buffer solution, using the data between 410 and 435 nm at each experimental temperature, gave the values of *k* and *l* from which the indicator ratio (*k*/*l*) was obtained. The values of *k*/*l* were used to calculate the equilibrium constants for ionization of DEHA using eq ([Disp-formula eq11]) and the resulting values for three sets of experiments are listed in Tables 2, 3, and 4, along with the statistical uncertainties, calculated from the standard deviation of the *k*/*l* vs λ mean. The effect of bias in the acid AcrH^+^ spectra is treated below in Section 4.3 as a systematic error. Figure 4 presents a plot of the experimental p*K*_a,H_ as a function of temperature.

**Table 2:** Experimental Values of the Ionization Constants of DEHA (Solution Set 1)

*t*/°C	p*K*_AcrH_^+^	*K*_AcrH_^+^	buffer ratio $\frac{\left[\mathrm{DEHA}\right]}{\left[\mathrm{DEHA}H^{+}\right]}$	indicator ratio $\frac{\left[\mathrm{Acr}H^{+}\right]}{\left[\mathrm{Acr}\right]}$	*Q*_a,H (DEHA)_ *= K*_a,H (DEHA)_ ^a^
50	5.019	9.55 × 10^–6^	0.8815	0.3981	(3.35 ± 0.04) × 10^–6^
75	4.697	2.00 × 10^–5^	0.8815	0.2932	(5.16 ± 0.06) × 10^–6^
100	4.423	3.80 × 10^–5^	0.8815	0.2435	(8.16 ± 0.05) × 10^–6^
125	4.187	6.46 × 10^–5^	0.8815	0.2486	(1.41 ± 0.02) × 10^–5^
150	3.983	1.05 × 10^–4^	0.8815	0.1281	(1.18 ± 0.02) × 10^–5^
200	3.655	2.19 × 10^–4^	0.8815	[0.2358]^b^	(4.55 ± 0.35) × 10^–5^
250	3.417	3.80 × 10^–4^	0.8815	0.1451	(4.86 ± 0.10) × 10^–5^

a The uncertainties (±) are the propagated experimental standard deviations of the parameters obtained from the regression.

b Strong dependence on λ, omitted from further data treatment.

**Table 3:** Experimental Values of the Ionization Constants of DEHA (Solution Set 2)

*t*/°C	p*K*_AcrH+_	*K*_AcrH_^+^	buffer ratio $\frac{\left[\mathrm{DEHA}\right]}{\left[{\mathrm{DEHAH}}^{+}\right]}$	indicator ratio $\frac{\left[{\mathrm{AcrH}}^{+}\right]}{\left[\mathrm{Acr}\right]}$	*Q*_a,H (DEHA)_ = *K*_a,H (DEHA)_ ^a^	Avg. *K*_**a,H (DEHA)**_ ^b^
50	5.019	9.55 × 10^–6^	0.1703	1.2618	(2.05 ± 0.01) × 10^–6^	(2.53 ± 0.24) × 10^–6^
			0.6016	0.4685	(2.69 ± 0.02) × 10^–6^	
			1.2926	0.2297	(2.84 ± 0.04) × 10^–6^	
100	4.423	3.80 × 10^–5^	0.1703	0.7274	(4.71 ± 0.03) × 10^–6^	(5.40 ± 0.35) × 10^–6^
			0.6016	0.2500	(5.72 ± 0.07) × 10^–6^	
			1.2926	0.1176	(5.78 ± 0.12) × 10^–6^	
150	3.983	1.05 × 10^–4^	0.1703	0.6953	(1.24 ± 0.01) × 10^–5^	(1.39 ± 0.08) × 10^–5^
			0.6016	0.2377	(1.50 ± 0.03) × 10^–5^	
			1.2926	0.1066	(1.44 ± 0.06) × 10^–5^	
200	3.655	2.19 × 10^–4^	0.1703	0.6598	(2.46 ± 0.02) × 10^–5^	(2.92 ± 0.23) × 10^–5^
			0.6016	0.2322	(3.06 ± 0.06) × 10^–5^	
			1.2926	0.1144	(3.23 ± 0.12) × 10^–5^	
250	3.417	3.80 × 10^–4^	0.1703	0.6882	(4.46 ± 0.03) × 10^–5^	(4.89 ± 0.22) × 10^–5^
			0.6016	0.2274	(5.20 ± 0.14) × 10^–5^	
			1.2926	0.1022	(5.02 ± 0.36) × 10^–5^	

a The uncertainties (±) are the propagated experimental standard deviations of the parameters obtained from the regression.

b The uncertainties (±) are the standard error of the mean.

**Table 4:** Experimental Values of the Ionization Constants of DEHA (Solution Set 3)

*t*/°C	p*K*_AcrH+_	*K*_AcrH+_	buffer ratio $\frac{\left[\mathrm{DEHA}\right]}{\left[\mathrm{DEHA}H^{+}\right]}$	indicator ratio $\frac{\left[\mathrm{Acr}H^{+}\right]}{\left[\mathrm{Acr}\right]}$	*Q*_**a,H (DEHA)**_ *= K*_**a,H (DEHA)**_ ^a^	Avg.*K*_**a,H (DEHA)**_ ^b^
25	5.397	3.98 × 10^–6^	0.2169	1.8801	(1.62 ± 0.01) × 10^–6^	(1.82 ± 0.20) × 10^–6^
			1.0976	0.4637	(2.03 ± 0.02) × 10^–6^	
50	5.019	9.55 × 10^–6^	0.2169	1.3457	(2.79 ± 0.01) × 10^–6^	(3.00 ± 0.30) × 10^–6^
			1.0976	0.3057	(3.20 ± 0.04) × 10^–6^	
100	4.423	3.80 × 10^–5^	0.2169	0.9094	(7.50 ± 0.03) × 10^–6^	(7.78 ± 0.28) × 10^–6^
			1.0976	0.1931	(8.06 ± 0.14) × 10^–6^	
150	3.983	1.05 × 10^–4^	0.2169	0.7747	(1.76 ± 0.04) × 10^–5^	(1.82 ± 0.06) × 10^–5^
			1.0976	0.1631	(1.87 ± 0.03) × 10^–5^	
200	3.655	2.19 × 10^–4^	0.2169	0.7702	(3.65 ± 0.02) × 10^–5^	(3.90 ± 0.24) × 10^–5^
			1.0976	0.1725	(4.14 ± 0.12) × 10^–5^	
250	3.417	3.80 × 10^–4^	0.2169	0.7903	(6.52 ± 0.06) × 10^–5^	(6.82 ± 0.31) × 10^–5^
			1.0976	0.1709	(7.13 ± 0.24) × 10^–5^	

a The uncertainties (±) are the propagated experimental standard deviations of the parameters obtained from the regression.

b The uncertainties (±) are the standard error of the mean.

**Figure 4:**
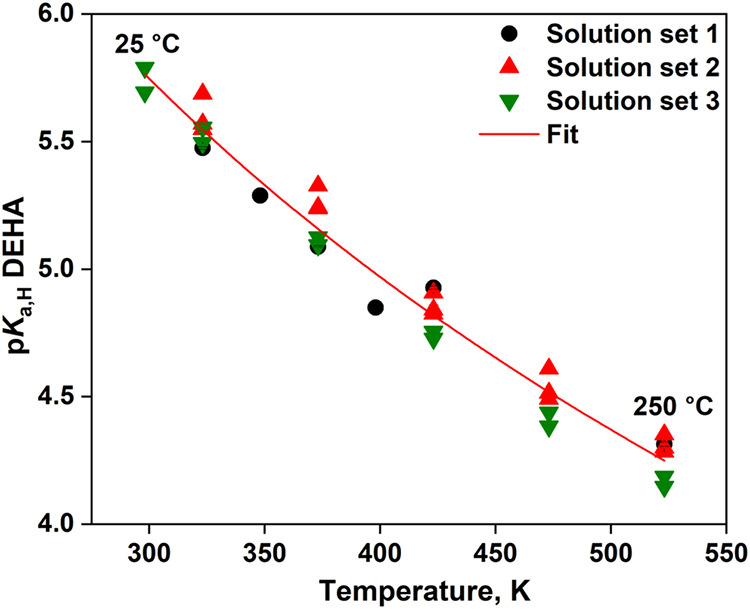
Temperature dependence of the ionization constant, p*K*_a,H_ of DEHA. Symbols represent experimental data points from Tables 2, 3 and 4. Line represents the fit of p*K*_a,H_ of DEHA using the extended van’t Hoff equation for an isocoulombic reaction, eq ([Disp-formula eq19]).

### 4.3 Thermochemical Models for the Ionization Constant of DEHA

The standard molar heat capacity of ionization, Δ_r_ *C*_*p*,298.15_^o^, for an isocoulombic reaction is often assumed to be independent of temperature^1,12^ and hence the temperature dependence of ionization constant can be represented by the extended van’t Hoff equation

$$\log K_{a,H,T}=\log K_{a,H,T_{r}}+\frac{\Delta_{r}{H_{T_{r}}}^{o}}{2.303R}[\frac{1}{T_{r}}-\frac{1}{T}]+\frac{\Delta_{r}{C_{p,T_{r}}}^{o}}{2.303R}\left[\ln\frac{T}{T_{r}}+\frac{T_{r}}{T}-1\right]$$ 19

To determine the thermochemical parameters log *K*_298.15_, Δ_r_ *H*_298.15_^o^ and Δ_r_ *C*_*p*,298.15_^o^, the experimental *K*_a,H_ data in Tables 2, 3 and 4 were regressed to eq ([Disp-formula eq19]) using the nonlinear regression algorithm in SigmaPlot and setting *T*_r_ = 298.15 K. The fitted thermochemical parameters thus obtained are reported in Table 5 and plotted in Figure 4.

**Table 5:** Thermochemical Parameters Obtained from Fitting Data in Tables 2, 3 and 4 Using eq ([Disp-formula eq19]) for the Isocoulombic Reaction: DEHAH^+^(aq)⇌DEHA­(aq) + H^+^(aq)

parameter	value^a^	standard error
log *K*_298.15_	–5.764	0.042
Δ_r_ *H*_298.15_^o^/(kJ mol^–1^)	15.50	2.00
Δ_r_ *C*_*p*_^o^/(kJ·K^–1^·mol^–1^)	0.050	0.019
p*K*_a,H_ (DEHA) at 25 °C	5.764	-
Standard error of log *K* fit	-	0.083

a These parameters correspond to eq ([Disp-formula eq20]), log *K*_a,H_ = −20.68–24.86/*T* + 6.06 log *T*.

The value p*K*_a,H_ = 5.764 ± 0.042 at 25 °C in Table 5 compares with the approximate value reported by Lim et al.,^11^ p*K*_a,H_ = ∼5.4 for *N*,*N*-diethylhydroxylamine which was estimated from apparent second-order rate constants for reactions with ozone measured at varying pH conditions.

The equilibrium constants were fitted with the simplified form of extended van’t Hoff equation for an isocoulombic reaction used in MULTEQ^1^ written as

20 $$\log K_{a,H,T}=a+\frac{b}{T}+c\cdot \log T$$

where *a*, *b*, and *c* are the fitting parameters. This fitted expression is mathematically identical to eq ([Disp-formula eq19]) with *T*_r_ = 0 K. In carrying out the regression, it was found that the parameter *b* was not statistically significant because the Δ_r_ *H*_0K_^o^[1/*T*] and Δ_r_ *C*_*p*,0K_^o^[*T*_r_/*T*] terms in eq ([Disp-formula eq19]) fortuitously cancel. The parameters *a* and *c* were then obtained by refitting the whole data set with the value of *b* set to zero. The fitting parameters obtained are listed in Table 6. The corresponding fit of experimental log *K*_a,H_ as a function of temperature is plotted in Figure S6.

**Table 6:** Fitting Parameters Obtained from the Three-Term Fit Using eq ([Disp-formula eq20]) to data in Tables 2, 3, and 4 for the Isocoulombic Reaction: DEHAH^+^(aq)⇌DEHA­(aq) + H^+^(aq)

parameter	value	standard error
*a*	–21.112	0.472
*b* [Table-fn t6fn1]	0	NA
*c*	6.20	0.18
p*K*_a,H_ (DEHA) at 25 °C	5.762	-
Standard error of log *K* fit	-	0.082

* The *b* parameter was found to be not statistically significant and was set to zero.

The difference plot of the fitted values of p*K*_a,H_ of DEHA obtained from the two-parameter and three-parameter fits using eq ([Disp-formula eq20]) relative to the fit using the van’t Hoff Equation, eq ([Disp-formula eq19]) is shown in Supporting Information (Figure S7). It is evident from the figure that the deviations are less than 0.002 in log *K*. The figure also includes a plot of experimental values of p*K*_a,H_ relative to the values from the van’t Hoff fit. The deviations in this case are less than 0.17, consistent with the experimental scatter.

The standard errors of the fitted parameters in eq ([Disp-formula eq19]) are lowest when the reference temperature *T*_r_ lies within the range of the experimental data, because it reduces the statistical covariance between the parameters. As a result, the standard errors of the fitted coefficients in eq ([Disp-formula eq20]) are large because the reference temperature *T*_r_ = 0 K lies so far beyond the experimental conditions. The values of log *K*_298.15_, Δ_r_ *H*_298.15_^o^ and Δ_r_ *C*_*p*,298.15_^o^ and their standard errors should be used in eq ([Disp-formula eq19]) to calculate these properties at other temperatures with physically meaningful propagated uncertainties. In addition to the statistical uncertainties calculated from eq ([Disp-formula eq19]) there is a systematic uncertainty associated with the acid spectra of AcrH^+^ discussed in Section 4.1. By artificially constraining the isosbestic points of all the spectra to the same value, as a “worst case”, we estimate that there is a systematic uncertainty of no more than 0.6 in log *K* at 250 °C and no more than 0.3 in log *K* at other temperatures, both in the positive direction. Measurements currently underway using other thermochemical methods suggest that this is an overestimate.

The equilibrium constants were also fitted with the “density” model^20^ given as

21 $$\log K_{a,H,T}=\left(a+\frac{b}{T}\right)+\left(f\cdot \log\rho_{w}\right)$$

where *a*, *b*, and *f* are the fitting parameters, and *ρ*_w_ is the density of water at the experimental temperature and pressure.^28,31^ Data from 200–250 °C in Tables 2, 3, and 4 were regressed to eq ([Disp-formula eq21]) to obtain the fitting parameter *f*. Parameters *a* and *b* were then obtained using the whole data set with the previously obtained *f* value held constant. The standard errors of the fitted parameters thus obtained were found to be higher than the regressions based on eqs ([Disp-formula eq19]) and ([Disp-formula eq20]). The fitted parameters are tabulated in Table 7.

**Table 7:** Fitting Parameters Obtained from the Mesmer “Density” Model Fit, eq ([Disp-formula eq21]) to Data in Tables 2, 3, and 4 for the Isocoulombic Reaction: DEHAH^+^(aq)⇌DEHA­(aq) + H^+^(aq)

parameter	value	standard error
*a*	–3.633	0.090
*b*	–617.3	35.2
*f*	–6.83	1.52
p*K*_a,H_ (DEHA) at 25 °C	5.714	
Standard error of log *K* fit	-	0.092

Although the density model yielded a less accurate fit to the experimental data, it can be used to predict pressure effects by substituting the appropriate values for the density of water in eq ([Disp-formula eq21]).

## 5 Discussion

### 5.1 Application to High-Temperature Chemical Equilibrium Models

This is the first reported study of ionization constants for *N*,*N*-diethylhydroxylamine (DEHA) at temperatures above 25 °C. Indeed, it is the first such high temperature study on any hydroxylamine. As was the case for previous systems studied, the UV–visible flow cell with acridine as a thermally stable pH indicator proved to be a powerful tool for measuring ionization constants at temperature higher than those accessible with a stirred pressure vessel equipped with yttria-zirconia, or other, pH electrodes, because the short residence times minimize the effect of thermal decomposition. Although the degree of decomposition observed at 250 °C prevented us from extending this study to higher temperatures, it is expected that extrapolations using the extended van’t Hoff eq (eqs 19 and 20) and density model fits (eq 21) will yield sufficiently accurate values for most steam generator applications to temperatures above 300 °C. The model substance activity coefficient model based on NaCl, eq ([Disp-formula eq10]), is valid over the range 125 to 275 °C and ionic strengths up to ∼1 mol·kg^–1^.^1,27^ These equations can be used directly in software such as MULTEQ and Chemworks-Tools (Electric Power Research Institute, Inc.), and in other chemical modeling tools, operated by the nuclear industry to model secondary coolant chemistry.

Equation ([Disp-formula eq19]) is recommended for calculating for these applications because its functional form provides the most accurate fit, and because the use of a constant heat capacity term, Δ_r_ *C*_*p*_^o^, has been found to yield accurate extrapolations for isocoulombic reactions.^12,20^ Statistical uncertainties for the extrapolated values of *K*_a,H_ can be calculated from the standard errors for the thermochemical parameters in eq ([Disp-formula eq19]), tabulated in Table 5. While the density model yielded a less accurate fit to the experimental data, it can be used to predict pressure effects by substituting the appropriate values for the density of water in eq ([Disp-formula eq21]). The pressure corrections to log *K*_a,H_ from 15 MPa to steam saturation pressure are within the standard errors of the fit by eq ([Disp-formula eq19]) over the experimental temperature range.

### 5.2 Implications for Steam Generator Chemistry

In addition to the quantitative results, this study yields some insights into the behavior of DEHA as a reducing agent to replace hydrazine additions for secondary coolant chemistry control. Although DEHA (p*K*_a,H_ = 5.764) is much more ionized at 25 °C than morpholine (p*K*_a,H_ = 8.491) and other alkalizing amines used for pH control, this difference is much less at 250 °C.^12,32,33^ This suggests that DEHA’s volatility will be similar to that of amines and decomposition products with similar molar mass under operating conditions. Although not a primary focus of this study, the measurable thermal decomposition observed at 250 °C, over the short time scales used in our experiments, suggests that the lifetime of DEHA in secondary coolant circuits may be short.

## 6 Summary and Conclusions

Together with the published literature on alkalizing amines, the work reported in this paper provides the ionization constants necessary to model the aqueous speciation of DEHA in nuclear reactor secondary coolant systems currently operating with hydrazine using chemical equilibrium models such as EPRI’s Chemworks Tools. The unusual temperature dependence of DEHA ionization relative to other amines, which is undoubtedly due to hydration effects on the –N–OH functional group, is also of fundamental interest. The rate of thermal decomposition was found to be significant above 250 °C. Further work to identify the decomposition reaction products and quantify the kinetics under these conditions would be desirable.

## Supplementary Material



## Notes

Selection of the pH indicator based on the model system (NH_2_–OH); ^1^H and ^13^C NMR spectra for experimental solutions; ^1^H and ^13^C NMR spectra for decomposition studies; additional fit for the ionization constant of DEHA using eq ([Disp-formula eq20]); difference between experimental data and fits using eq ([Disp-formula eq20]) relative to p*K*_a,H(Fit eq (19))_; Raman spectra for experimental solutions and peak fitting to calculate the extent of decomposition (PDF)
