# Supplementary material for: Experimental Determination of the Ionization Constant of N,N‑diethylhydroxylamine (DEHA) from 25 to 250 °C at 15 MPa
Source: J Chem Eng Data. 2026 Jul 30;71(8):3372–80. doi: 10.1021/acs.jced.6c00121 (PMC13479606; doi:10.1021/acs.jced.6c00121)
Supplement: Supplementary file 1 [file je6c00121_si_001.pdf]

Supplementary Information

for

Experimental Determination of the Ionization Constant of N,N-diethylhydroxylamine (DEHA) from 25 to 250 °C at 15 MPa

Sreedhanya Swaroop, Swaroop Sasidharanpillai and Peter R. Tremaine*

Department of Chemistry, University of Guelph,
50 Stone Rd. E., Guelph, ON,
Canada N1G 2W1.

*Corresponding author's email address: tremaine@uoguelph.ca

S1. UV-Visible Spectroscopic Scoping Study with Model System NH_2OH

The ionization constants $K_{a,H}$ for DEHA were measured at temperatures from 25 to 250 °C, using acridine as the colorimetric indicator. Acridine was selected as the suitable indicator for DEHA system based on initial calculations using the van't Hoff equation with constant ΔC_p^o to extrapolate the value of log $K_{a,H}$ = -5.4 for DEHA at 25 °C[1] to our experimental conditions. These were done by taking NH_2-OH as model system, using values of $\Delta_{ion}H^o_{298}$ or $\Delta_{ion}C_p{}^o{}_T$ reported by Lumme et al.[2] since the values for DEHA are not available in the literature.

The values of of p$K_{a,AcrH^+}$ = -log $K_{a,AcrH^+}$ (Ryan et al.)[3] and p$K_{a,\ \beta\text{-NA}}$ = -log $K_{a,\ \beta\text{-NA}}$ (Xiang and Johnston)[4] plotted along with the values for NH_2OH extrapolated from room temperature data are presented in Figure S1. The region within log K_{HIn^+} ± 1 corresponds to the expected practical limits of meaningful measurements where measurable concentrations of ionized and neutral forms of the indicator are present. Based on the results, acridine was found to be suitable for temperatures ranging from 25 - 250 °C.

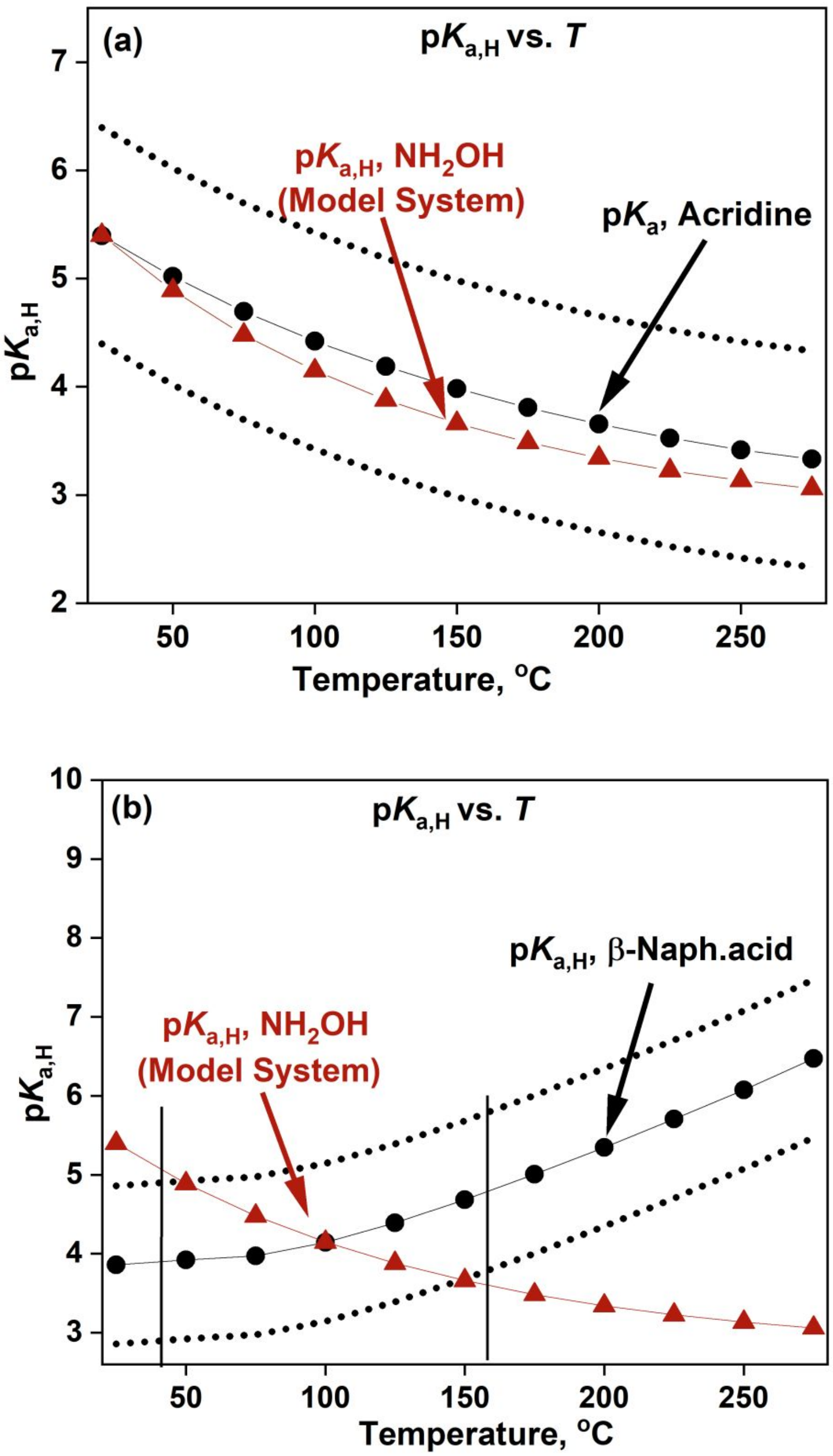

Figure S1. The temperature dependance of $pK_{a,H}$ for NH_2-OH (solid red triangles), estimated from room temperature thermodynamic data using van't Hoff equation. Solid black circles represent $pK_{a,H}$ of the indicators: acridine (a) and β-naphthoic acid (b). Dotted lines represent the indicator range defined as $-\log K_{HIn^+} \pm 1$. The vertical solid lines show the temperature range suitable for the use of β-naphthoic acid as indicator for NH_2-OH system.

S2: Thermal Decomposition Studies: NMR Analysis

S2.1 NMR Analysis of Experimental Solutions

In our scoping studies, the effect of flow rate on the UV-visible spectra of the DEHA buffer solutions was examined. Above 150 °C, the spectra showed spectral intensity changes at a flow rate of 0.1 $mL.min^{-1}$. The UV-visible spectra of the experimental solutions recorded at a flow rate of 1.0 $mL.min^{-1}$ showed no signs of decomposition (constant spectrum) even at 250 °C.

In studies to determine the decomposition reaction products for NMR analysis, the experimental flow rate was set at 0.1 $mL.min^{-1}$ to increase the residence time of the solution at elevated temperature, and at 1.0 $mL.min^{-1}$ to minimize the residence time of the solution at elevated temperature to minimize thermal decomposition above 150 °C.

Thermal decomposition studies using NMR to examine the thermal decomposition products of DEHA solutions were carried out at two pump flow rates (1.0 $mL.min^{-1}$ and 0.1 $mL.min^{-1}$) at 250 °C, the highest temperature studied. The pressure was set to 15 MPa, high enough above the boiling point of water to guarantee that only the liquid phase was present under all the experimental conditions. Samples of the original un-heated solution, and quenched samples of heated solutions that had passed through the cell, were spiked with sodium 2,2-dimethyl-2-silapentane-5-sulfonate (DSS) as an NMR internal standard for quantification and analysis by ^{1}H NMR and ^{13}C NMR.

Figure S2 represents the ^{1}H NMR spectra of DEHA buffer solutions before and after heating at 250 °C in the UV-visible flow cell at a flow rate of 1.0 $mL.min^{-1}$ and 0.1 $mL.min^{-1}$. Figure S2(a) for the un-heated sample shows the characteristic DEHA peaks centered around 1.17 ppm (triplet, $-CH_3$ proton); 3.02 - 3.15 ppm (double quartet peak, $-CH_2$ proton) and that of the internal standard (DSS) at -0.08 ppm (singlet). The spectrum for the sample run at 1.0 $mL.min^{-1}$ at 250 °C is shown

in Figure S2(b). It is evident that DEHA remained almost unchanged with minimal decomposition under these conditions. The percentage decomposition of DEHA is estimated to be 6.87 ± 1.75 % from the ^{1}H NMR peak integrated peak areas relative to the DSS standard.

The ^{1}H NMR spectrum of the quenched solution from the experimental run at 250 °C with longer residence time, 0.1 $mL.min^{-1}$, is shown in Figure S2(c). The intensities of the DEHA peaks were significantly reduced, and the appearance of small new peaks at approximately 2.1, 1.88 and 1.24 ppm indicated the presence of thermal decomposition of products under these conditions. Based on the integrated ^{1}H band areas, the percentage decomposition of DEHA is estimated to be 21.7 ± 1.8 %. Based on these results, the thermal decomposition of DEHA in the experimental solution run at 1.0 $mL.min^{-1}$ at 225 °C is estimated to be ~ 2%.

Quantitative analysis using the ^{13}C NMR spectra using the DSS internal standard, yielded more limited results, due to the much lower sensitivity of the method. However, spectra were obtained for DEHA buffer solutions from the high temperature cells using a flow rate of 0.1 $mL.min^{-1}$ at both 225 and 250 °C. Figure S3 shows the ^{13}C NMR spectra of the un-heated DEHA buffer solutions, and after heating in the UV-visible flow cell using a flow rate of 0.1 $mL.min^{-1}$ at 225 °C and 250 °C. The spectrum of the un-heated sample in Figure S3(a) displays characteristic DEHA peaks at ~8.8 ppm ($-CH_3$ carbon) and 53.8 ppm ($-CH_2$ carbon). The spectrum of quenched solution after 225 °C run in Figure S3 (b) does not show the presence of any significant decomposition products. At 250 °C and a flow rate of 0.1 $mL.min^{-1}$, additional peaks corresponding to the lower alkyl groups (~23.23 ppm) and other peaks around 42, 35 and 12 ppm were observed in the spectrum shown in Figure S3 (c). This is consistent with the ^{1}H NMR results. Both the ^{1}H and ^{13}C NMR analyses show that the thermal decomposition of DEHA in the UV-visible flow cell at a flow rate of 1.0 $mL.min^{-1}$ is minor, ≤ 2 % at 225 °C and ~ 7 % at 250 °C.

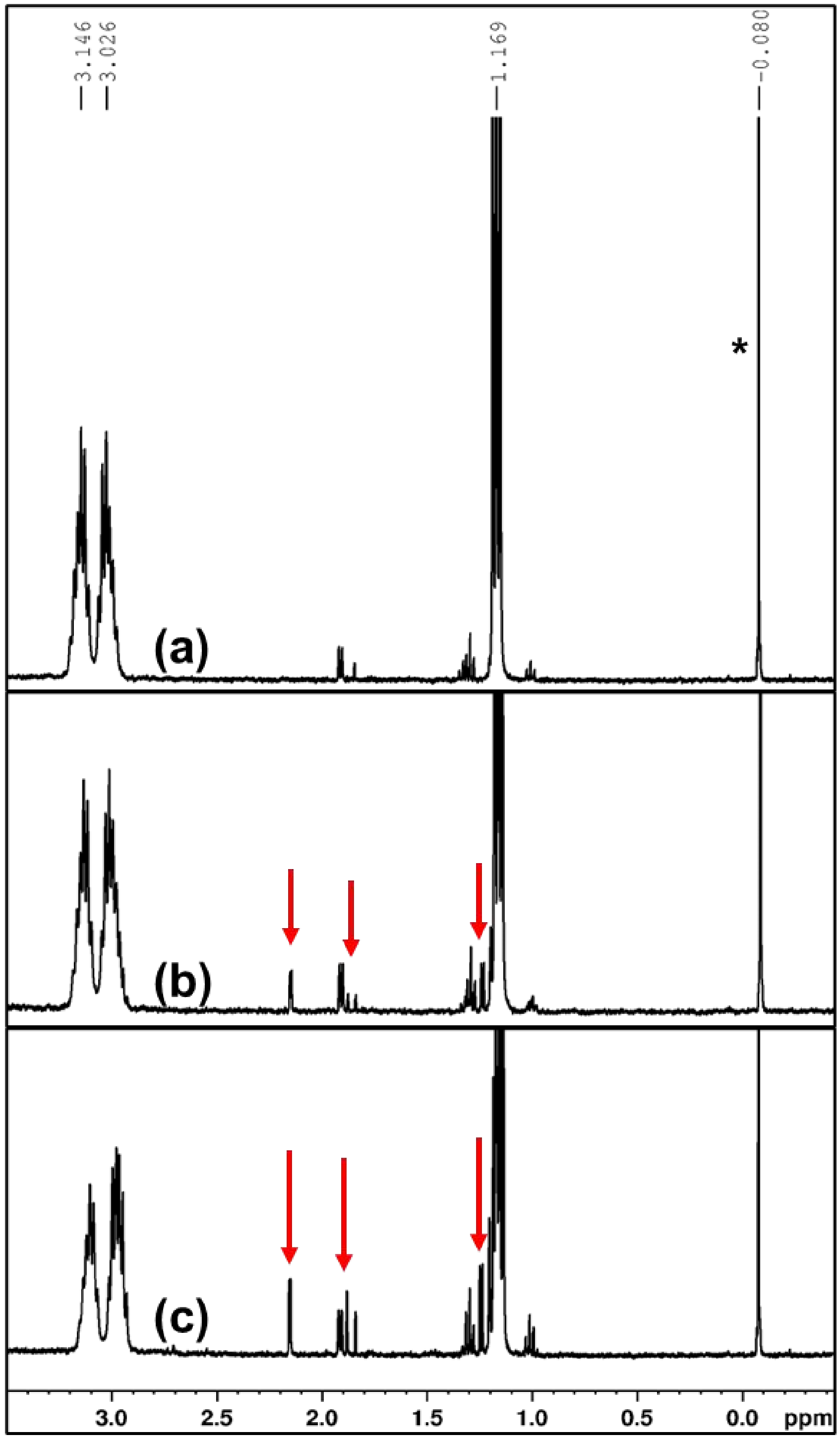

Figure S2. ^1H NMR spectra of DEHA buffer solutions: (a) original solution 25 °C before injection; (b) quenched solutions from 250 °C run at flow rate 1.0 $mL.min^{-1}$; (c) quenched solutions from 250 °C run at flow rate 0.1 $mL.min^{-1}$. The red arrows show the NMR peaks corresponding to the decomposition products and the asterisk represents the DSS (internal standard) peak.

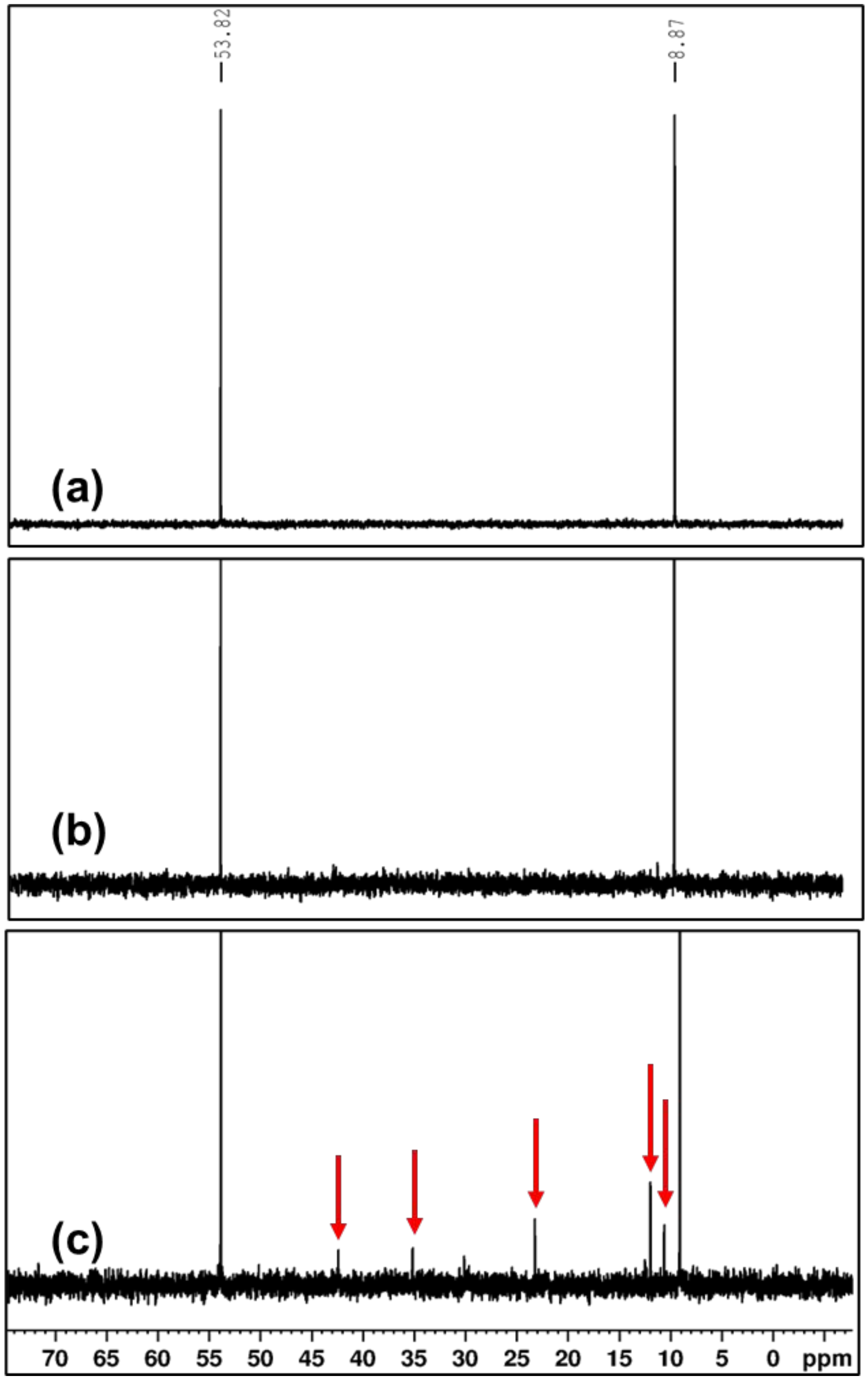

Figure S3. ^{13}C NMR spectra of DEHA buffer solutions: (a) original solution 25 °C before injection; (b) quenched solutions from 225 °C run at flow rate 0.1 mL.min^{-1}; (c) quenched solutions from 250 °C run at flow rate 0.1 mL.min^{-1}. The red arrows show the NMR peaks corresponding to the decomposition products. Spectra (b) and (c) are scaled for better visualization of the decomposition product peaks.

S2.2 Thermal Decomposition Products of DEHA

Absence of any appreciable change in NMR spectra (Figure S2b) suggested minimal thermal decomposition of DEHA under the UV-visible experimental conditions. To identify the thermal decomposition products of DEHA, a high temperature experiment was carried out in a Teflon lined Paar 4744 autoclave cell in order to increase the concentrations of the decomposition products to the level needed for unambiguous ^{13}C and ^{1}H NMR spectra. The spectra were obtained from an aqueous 0.21 mol.kg^{-1} solution of DEHA that was heated for 1 hour at 250 °C in a Teflon lined Paar 4744 autoclave cell, then quenched rapidly in ice water. The quenched solution was analyzed using NMR spectroscopy.

The ^{1}H NMR spectrum of 0.21 mol.kg^{-1} DEHA solution before heating at 250 °C is shown in Figure S4a. The spectrum reveals the characteristic DEHA peaks centered around 1.05 ppm (triplet, -CH_3 proton) and 2.73 – 2.88 ppm (double quartet peak, -CH_2 proton). The corresponding NMR spectra for DEHA solutions after heating at 250 °C (Figure S4b) revealed the characteristic DEHA peaks along with the following additional peaks at: δ = 0.99 (t), 1.00 (t), 1.05 (t), 1.28 (t), 1.81 (s), 1.86 (s), 1.97 (s), 2.64, 2.69, 3.07 and very small peaks around 3.75 and 7.26 ppm.

The ^{13}C NMR spectrum of the DEHA solution before and after heating at 250 °C is given as Figure S5a and b. In addition to the characteristic DEHA peaks at 9.86 ppm (-CH_3 carbon) and 53.88 ppm (-CH_2 carbon); additional peaks around δ = 11.84, 12.28, 12.47, 13.40, 21.89, 34.59, 42.54, 59.11, 144.18, 173.82 and 181.43 ppm (phased down) were observed in the spectrum (Figure S5b).

Previous thermal decomposition studies of DEHA under boiler conditions showed that diethylamine, ethylmethylamine acetaldehyde and acetate/acetic acid were the major products,

whereas acetaldoxime was found to be the major product during the low-pressure studies along with ammonia as a minor product[5].

The presence of triplet peaks around 0.99 and 1.00 ppm (-CH_3 proton) along with the quartets around 2.64 and 2.69 ppm (-CH_2 proton) in Figure S4b suggests the formation of diethylamine and methylethylamine from DEHA at 250 °C, consistent with the literature[5]. The peaks around 13.40 (-CH_3 carbon) and 34.59 ppm (-CH_2 carbon) present in the ^{13}C spectrum (Figure S5b) further confirms the formation of DEA and MEA.

The ^{1}H and ^{13}C NMR spectra of the DEHA solution after heating at 250 °C for 1 hour confirmed the presence of DEA and MEA along with acetic acid/acetate. It is also suggested that the presence of N-ethylethanamide or N-ethyl-N-hydroxyacetamide is likely.

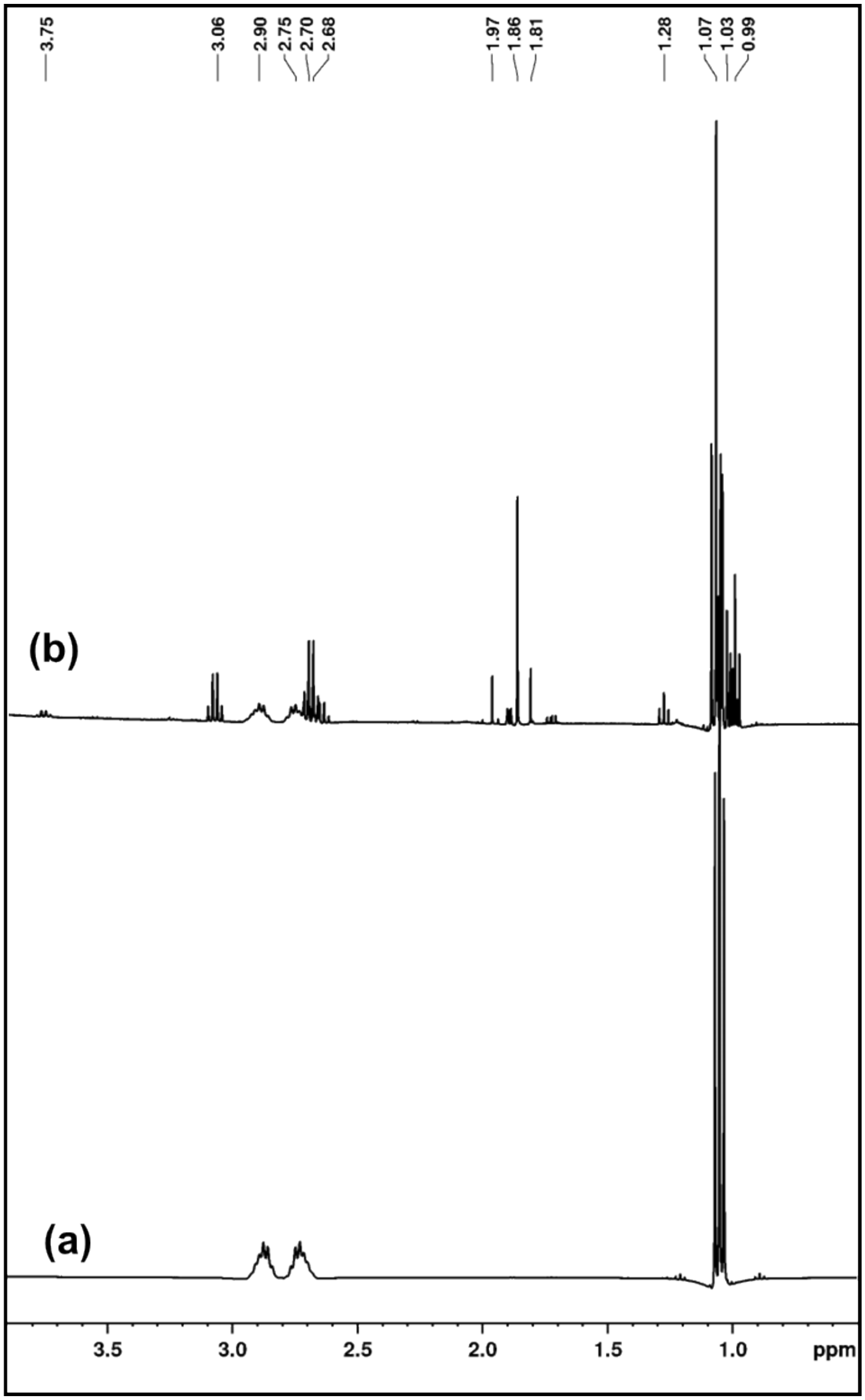

Figure S4. ^{1}H NMR spectra of 0.21 mol.kg^{-1} DEHA solutions: (a) before heating; (b) quenched solutions after heating at 250 °C for 1 hour. Spectra are scaled for better visualization of the decomposition product peaks.

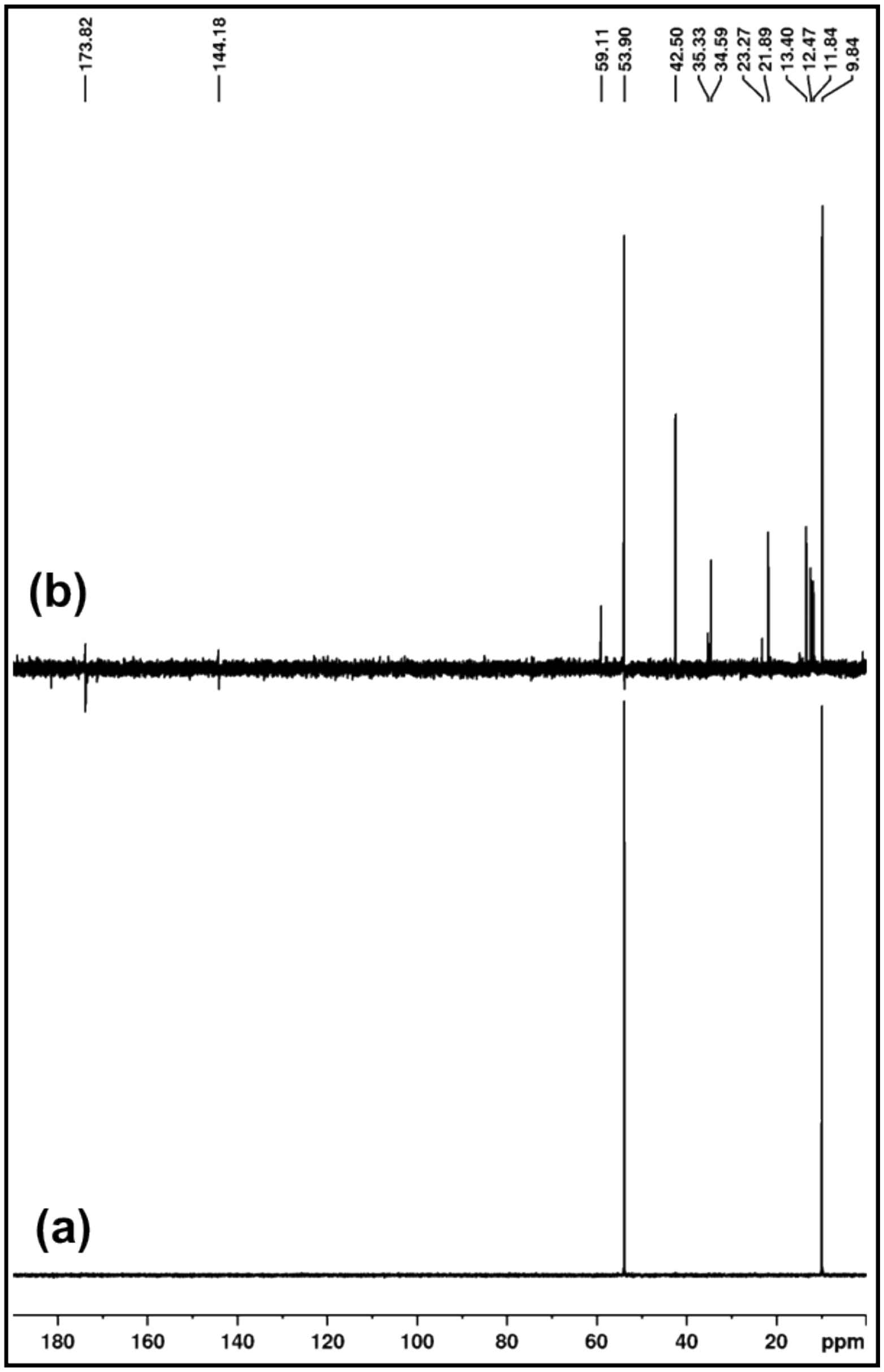

Figure S5. ^{13}C NMR spectra of 0.21 mol.kg^{-1} DEHA solutions: (a) before heating; (b) quenched solutions after heating at 250 °C for 1 hour. Spectra are scaled for better visualization of the decomposition product peaks.

S3: Other Thermochemical Models for the Ionization Constant of DEHA

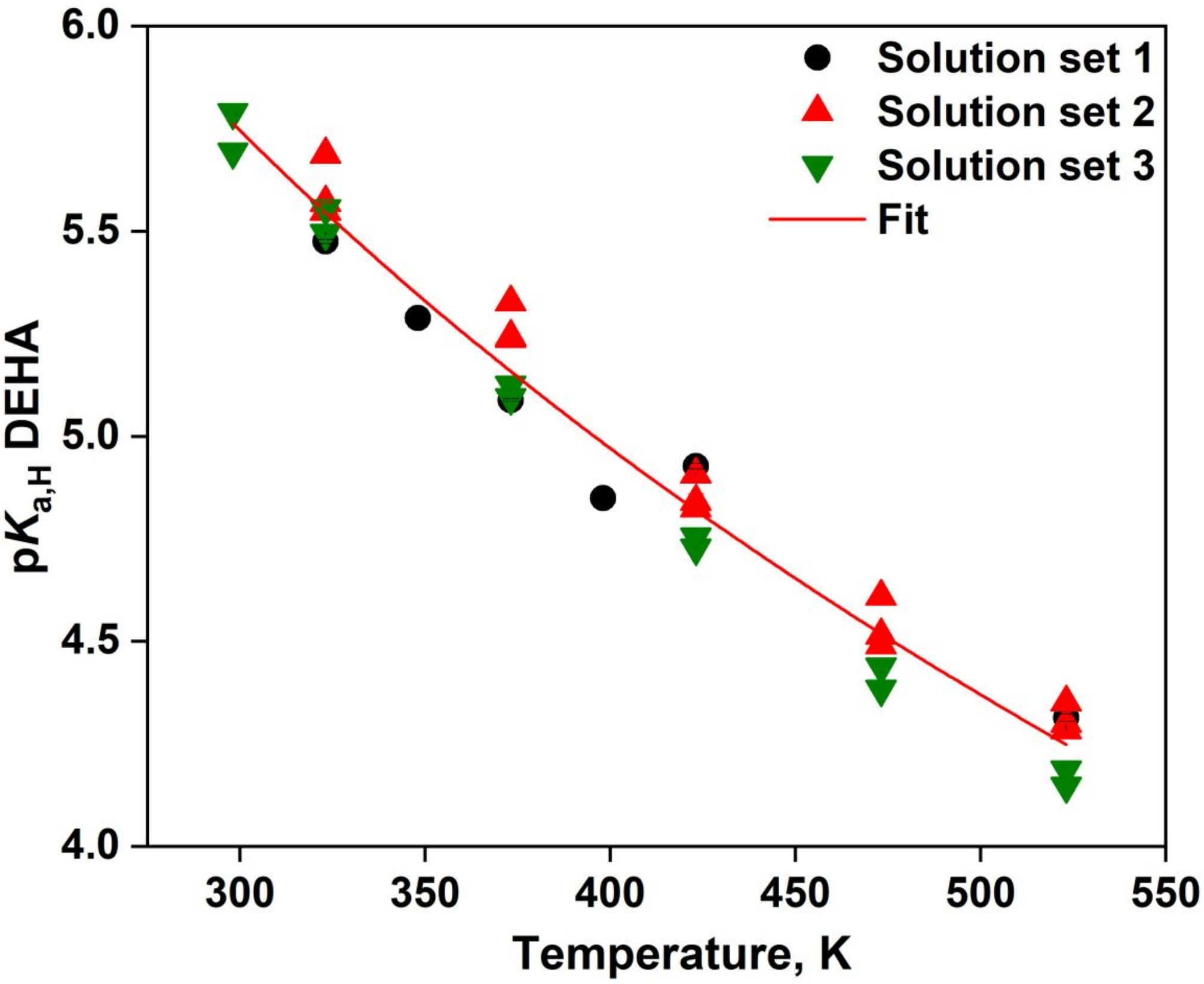

Figure S6. Temperature dependence of the ionization constant, p$K_{a,H}$ of DEHA. Symbols represent experimental data points from Tables 2, 3 and 4. Line represents the fit of p$K_{a,H}$ of DEHA using the simplified form of the extended van't Hoff equation for an isocoulombic reaction, Eq. (20) with the *b* parameter set to zero.

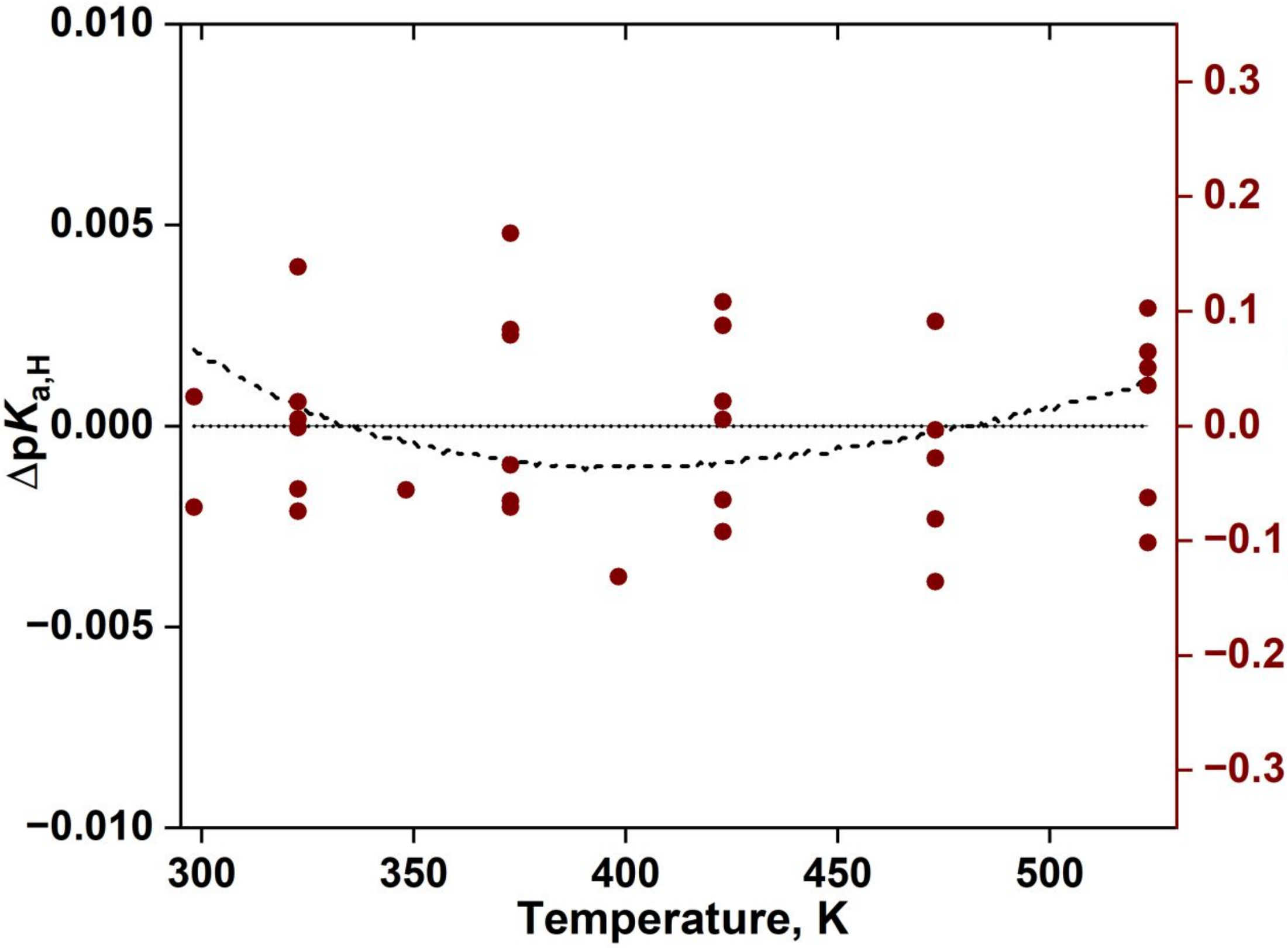

Figure S7. Deviation plot showing the difference between experimental data and fit with two and three parameters using Eq. (20) relative to the van't Hoff fit using Eq. (19). Symbols represent experimental values ($pK_{a,H(Expt)} - pK_{a,H(Fit)}$); dashed line represents fit with the b parameter set to zero ($pK_{a,H(Fit\ Eq.\ (20))} - pK_{a,H(Fit)}$); dotted line represents fit with a, b and c parameters ($pK_{a,H(Fit\ Eq.\ (20))} - pK_{a,H(Fit)}$) relative to the values from van't Hoff fit.

S4: Thermal Decomposition Studies: Raman Spectroscopic Analysis

Raman spectra of DEHA solutions were recorded before and after the UV-visible spectral acquisition at 250 °C to estimate the extent of thermal decomposition during the spectral acquisition process at two different flow rates (1.0 and 0.1 $mL.min^{-1}$). These spectra were compared with the Raman spectrum of the same solution before the UV-visible experiment. Figure S8 shows the baseline corrected Raman spectra of (a) the solution at 25 ^{0}C before injection, (b) quenched solutions from 250 ^{0}C run at 1.0 $mL.min^{-1}$ and (c) 0.1 $mL.min^{-1}$. The background levels in the spectra for the quenched solution flow rate 1.0 $mL.min^{-1}$ was higher than the background of the unheated solution and that of the solution run at lower flow rate (0.1 $mL.min^{-1}$) was even higher. These are consistent with the NMR results discussed above, indicating thermal decomposition at 250 °C. Both quenched solutions from 250 ^{0}C runs (Figure S8: b and c) has Raman peaks corresponding to DEHA even though there are changes in the intensity and background level.

Figure S9 depicts the deconvoluted peaks of 1450 cm^{-1} (CH_3/CH_2 deformation) region of spectra shown in Figure S8. There is a slight decrease in the intensity of DEHA peak at 1450 cm^{-1} with the appearance of new peaks around 1400 and 1490 cm^{-1} in the spectra for the solution ran at 1.0 $mL.min^{-1}$ (b), compared to the starting solution (a). For the solution ran at 0.1 $mL.min^{-1}$(c), the intensity of the DEHA peak decreased further and the intensity of new peaks were higher. The thermal decomposition of DEHA in the 0.1 $mL.min^{-1}$ run is evident from the decrease in intensity of the 1450 cm^{-1} peak and the appearance of the additional peaks from the decomposition products, whereas the extent of decomposition is minimal in the solution ran at 1.0 $mL.min^{-1}$. These results are consistent with the results from quantitative ^{1}H NMR analysis.

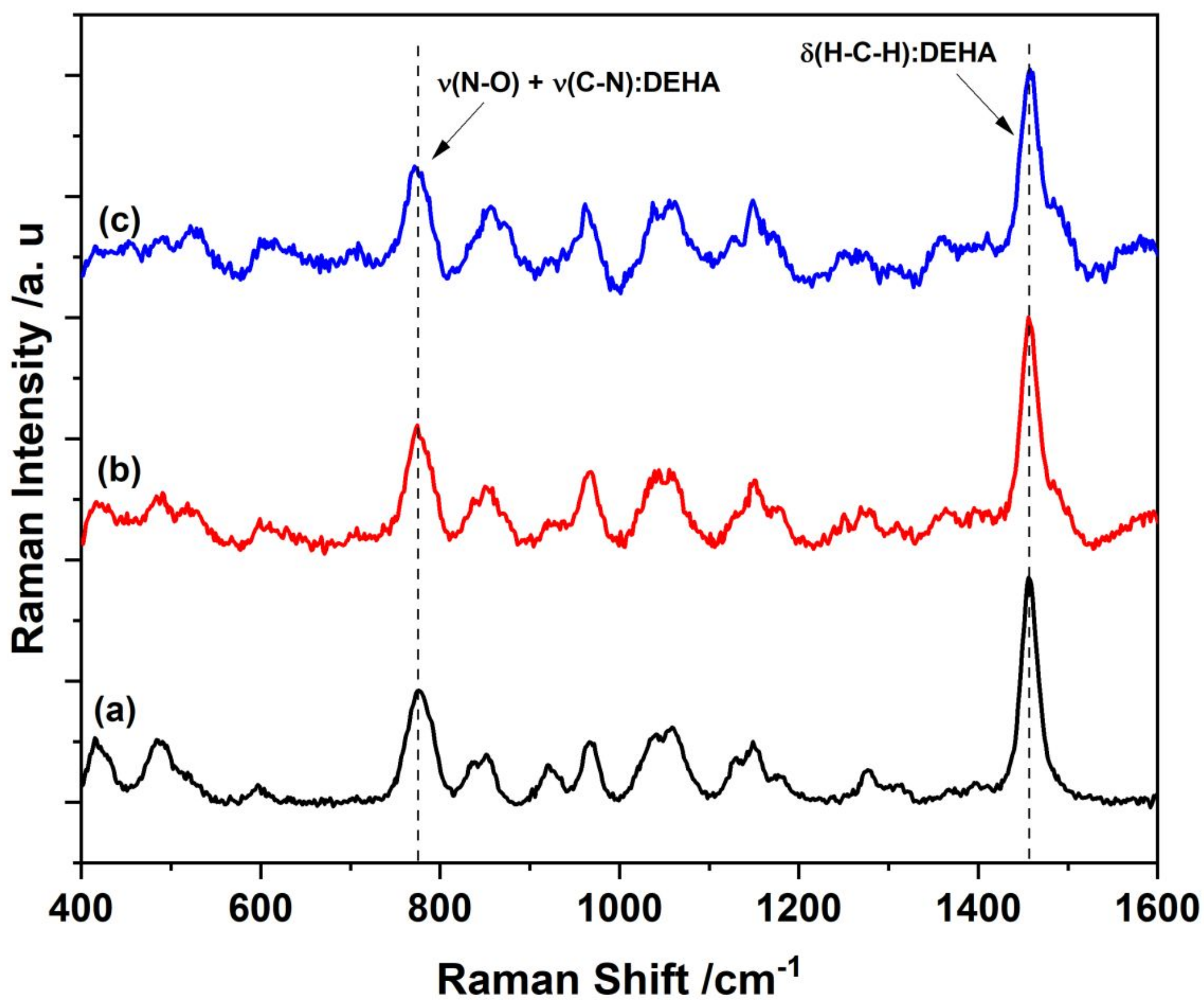

Figure S8. Baseline-corrected Raman spectra Raman spectra at 25 °C of quenched 0.12 mol.kg^{-1} DEHA buffer solutions from UV-visible experimental runs showing the change in background of the spectra: (a) original solution 25 °C before injection; (b) quenched solutions from 250 °C run at flow rate 1.0 mL.min^{-1}; (c) quenched solutions from 250 °C run at flow rate 0.1 mL.min^{-1}.

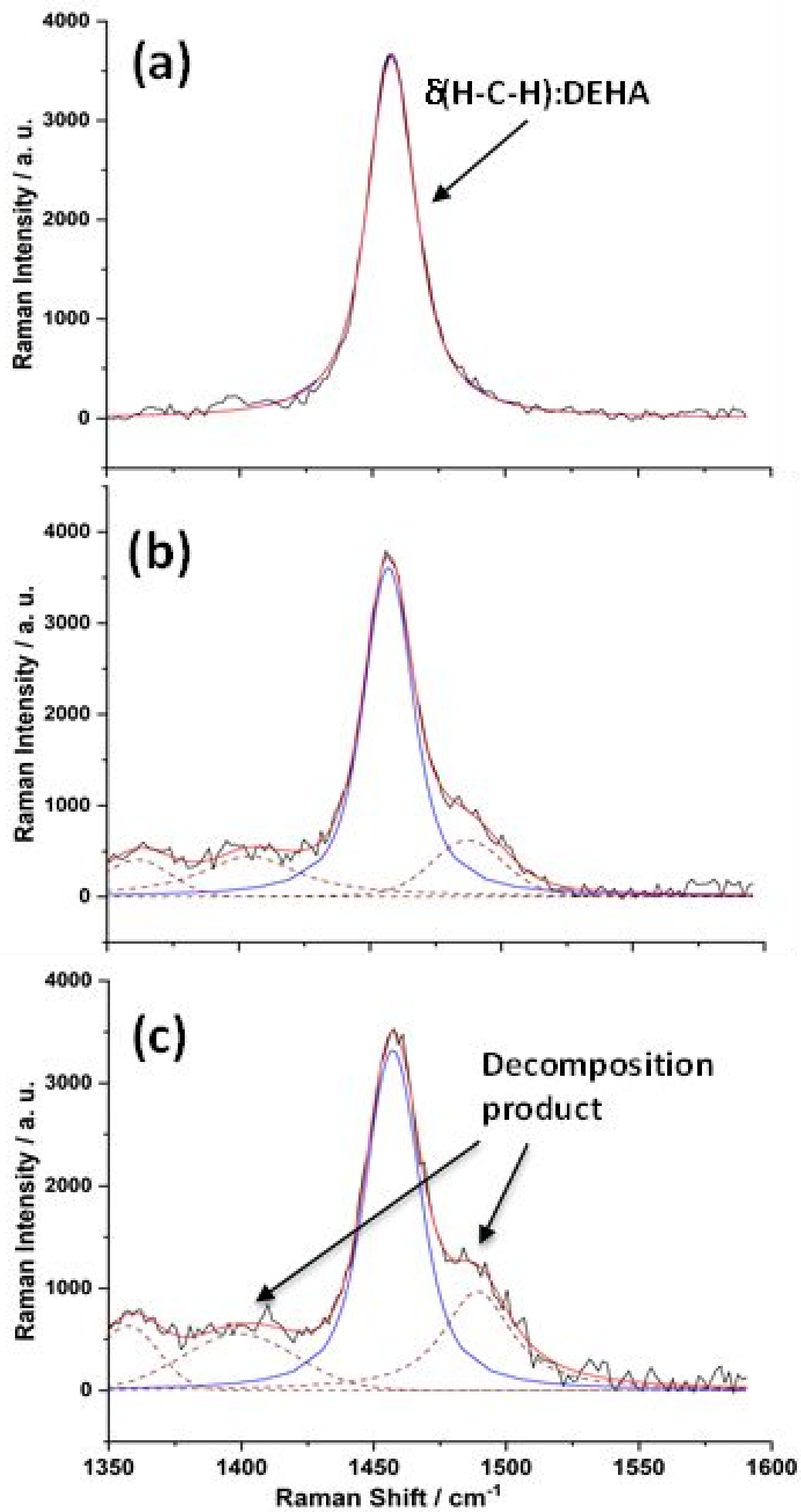

Figure S9. Baseline-corrected Raman spectra in the 1350-1600 cm^{-1} range of quenched 0.12 mol.kg^{-1} DEHA buffer solutions at 25 °C from Figure S9b, showing the peak fit used to estimate the relative change in concentration of DEHA and the appearance of bands due to decomposition products: (a) original solution 25 °C before injection; (b) quenched solutions from 250 °C run at flow rate 1.0 mL.min^{-1}; (c) quenched solutions from 250 °C run at flow rate 0.1 mL.min^{-1}.

S5: References

(1) Lim, S.; McArdell, C. S.; von Gunten, U. Reactions of aliphatic amines with ozone: Kinetics and mechanisms. *Water Research* **2019**, *157*, 514-528.

(2) Lumme, P.; Lahermo, P.; Tummavuori, J. Thermodynamics of the ionization of hydroxylamine and nitrous acid in water. *Acta Chem. Scand* **1965**, *19* (9).

(3) Ryan, E. T.; Xiang, T.; Johnston, K. P.; Fox, M. A. Absorption and Fluorescence Studies of Acridine in Subcritical and Supercritical Water. *The Journal of Physical Chemistry A* **1997**, *101* (10), 1827-1835.

(4) Xiang, T.; Johnston, K. P. Acid-base behavior in supercritical water: β-naphthoic acid-ammonia equilibrium. *Journal of Solution Chemistry* **1997**, *26* (1), 13-30.

(5) Marks, C. *Hydrazine Alternatives for the Pressurized Water Reactor/Pressurized Heavy Water Reactor Secondary System: Diethylhydroxylamine for Operational Use.* ; Electric Power Research Institute Palo Alto, CA, 2021.
